# Impact of Species, Growth Conditions, and Plant Processing on the Phytochemistry and Antimicrobial Activity of *Agrimonia* Extracts

**DOI:** 10.1002/cbdv.202501283

**Published:** 2025-08-08

**Authors:** Anna Loučková, Jarmila Neugebauerová, Bára Křížkovská, Marie Zlechovcová, Kateřina Šebelová, Jan Lipov, Jitka Viktorová, Jana Hajšlová

**Affiliations:** ^1^ Department of Food Analysis and Nutrition, Faculty of Food and Biochemical Technology The University of Chemistry and Technology, Prague (UCT Prague) Prague Czech Republic; ^2^ Department of Growing Vegetable and Floriculture, Faculty of Horticulture Mendel University in Brno (MENDELU) Lednice na Moravě Czech Republic; ^3^ Department of Biochemistry and Microbiology, Faculty of Food and Biochemical Technology The University of Chemistry and Technology, Prague (UCT Prague) Prague Czech Republic

**Keywords:** agrimony, antimicrobial activity, liquid chromatography, mass spectrometry, phytochemistry, polyphenols

## Abstract

*Agrimonia eupatoria* L., a herb used in traditional medicine, contains numerous secondary metabolites with beneficial properties. However, its phytochemistry, and consequently bioactivity, can be strongly influenced by various cultivation and processing factors. This study evaluates the impact of growth locality, plant part, ontogenetic phase, and postharvest processing on the phytochemical composition and antimicrobial potential of two agrimony species: *A. eupatoria* and *Agrimonia procera*. A total of 94 herbal samples extracted with 40% aqueous ethanol were analyzed using ultrahigh‐performance liquid chromatography and high‐resolution tandem mass spectrometry and simultaneously tested against selected Gram‐positive, Gram‐negative, and anaerobic bacteria and yeasts. Targeted screening of bioactive polyphenols revealed significant differences between the agrimony species, with phloridzin and orientin/isoorientin identified as new potential chemotaxonomic markers. Notable differences were also observed between roots and aerial parts. Postharvest processing, particularly drying, proved to be another important factor, reducing the overall polyphenol content compared to fresh‐frozen plant material. Although all extracts exhibited strong activity, especially against Gram‐positive bacteria, the highest antimicrobial potential was observed in roots extracts rich in procyanidins and catechins. The findings of this study enhance the understanding of agrimony phytochemistry and its variability, thereby supporting the effective production of medicinal preparations with the desired therapeutic effects.

## Graphical abstract

Agrimony is a medicinal herb known for its content of polyphenols and beneficial bioactivity, both of which can be strongly influenced by cultivation and processing. This study reveals that agrimony plant structures, species, and postharvest processing are the main drivers of observed variability in both phytochemical composition and antimicrobial potential.

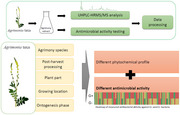

## 1 Introduction

Due to their many beneficial effects on human health, medicinal herbs have been used in various forms for ages. One of the herbs widely used against respiratory tract infections in traditional medicine is common agrimony (*Agrimonia eupatoria* L.), a perennial plant from the Rosaceae family. Agrimony contains a variety of bioactive compounds and such as pose a wide range of biological activities [1]. However, its bioactive compound profile is influenced by many factors and, thus, varies among plants. Still little is known about how this variability influences its biological activities and the efficacy of its medicinal preparations.

Among the various secondary metabolites reported for agrimony (e.g., triterpenoids, terpenes, and organic acids), polyphenols, including flavonoids, phenolic acids, and tannins, are dominating. They show a number of biological activities, including antioxidant, antiviral, hepatoprotective, and/or antimicrobial effects [2, 3, 4, 5]. The polyphenol profiles of agrimony plants have been investigated within various studies [2, 4, 6, 7, 8, 9, 10], a detailed review of which has been published recently by Malheiros et al. [3]. One of the most extensive studies focused on agrimony species was published by Granica et al. who qualitatively analyzed polyphenols profiles in eight *A. eupatoria* L. and five *Agrimonia procera* WALLR. samples obtained both from commercial manufacturers and from nature [6]. On the basis of their results, vitexin and isovitexin were proposed as chemotaxonomic markers for distinguishing these two species. Karlińska et al. characterized quantitative differences between the polyphenol profiles of *A. eupatoria* L. and *A. procera* WALLR. during different vegetation periods and described polyphenols variability among different plant parts [10]. Kubínová et al. investigated anticholinesterase and antioxidant activities of aqueous extracts of five agrimony species (*Agrimonia coreana* NAKAI, *Agrimonia japonica* KOIDZ, *A. procera* WALLR., *A. eupatoria* L., and *Agrimonia leucantha* KUNZE) and reported that the latter activity was most intensive in *A. procera* WALLR. characterized by the highest polyphenol content [11]. A couple of studies also documented that biological activities of agrimony extracts, such as antioxidant activity, depend mainly on polyphenol content [10, 12, 13]. Despite all of these studies, the influence of other important factors, such as plant growth locality and sample postharvest processing, on the polyphenolic profiles of agrimony species and the biological activities of agrimony extracts has been overlooked so far. Filling this knowledge gap can significantly improve the understanding of the phytochemical and biological potential of agrimony taxa, what is an essential prerequisite for the effective cultivation and processing of this valuable herb and the subsequent development of preparations with desired therapeutic effects.

Various strategies are currently employed in pharmaceutical industry for isolation of bioactive compounds from natural plant materials [14]. In the case of agrimony, traditional preparations include aqueous extracts (infusions) and aqueous ethanol extracts (tinctures), typically made from the aerial parts of the plant.

Within this study, we applied a complex strategy involving ultrahigh‐performance liquid chromatography and high‐resolution tandem mass spectrometry (UHPLC–HRMS/MS) analysis, followed by bioactivity testing, with the aim to assess differences between the phytochemical profiles and antimicrobial activities of hydroalcoholic extracts of various agrimony samples. To provide the most comprehensive study to date, we focused not only on the impact of agrimony plant species, plant part, and ontogenetic phase, but also on that of growth locality and postharvest sample processing (drying/fresh‐freezing), as factors whose impact has not been reported yet.

## 2 Results and Discussion

To explore the phytochemical composition of tested agrimony extracts and its variability resulting from various cultivations and processing conditions, statistical analysis of the non‐targeted UHPLC–HRMS/MS data followed by targeted (suspect) screening of polyphenols was carried out. On the basis of the obtained results, we evaluated the impact of agrimony species, growth locality, ontogenetic (developmental) phase, and postharvest processing. Antimicrobial activity of the corresponding agrimony extracts was also assessed and subsequently correlated with the detected polyphenolic compounds.

### 2.1 Significance of Factors Affecting the Phytochemical Profile of Agrimony

Principal component analysis (PCA) of non‐targeted UHPLC–HRMS/MS metabolomic data was performed to assess differences in total phytochemical profiles among the agrimony samples and determine the factors with the highest impact.

**FIGURE 1:**
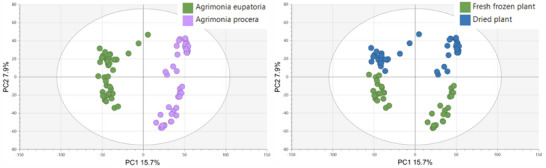
Principal component analysis (PCA) score plots (PC1 and PC2) of the agrimony aerial parts clustered according to species and postharvest sample processing (drying vs. fresh‐freezing).

**FIGURE 2:**
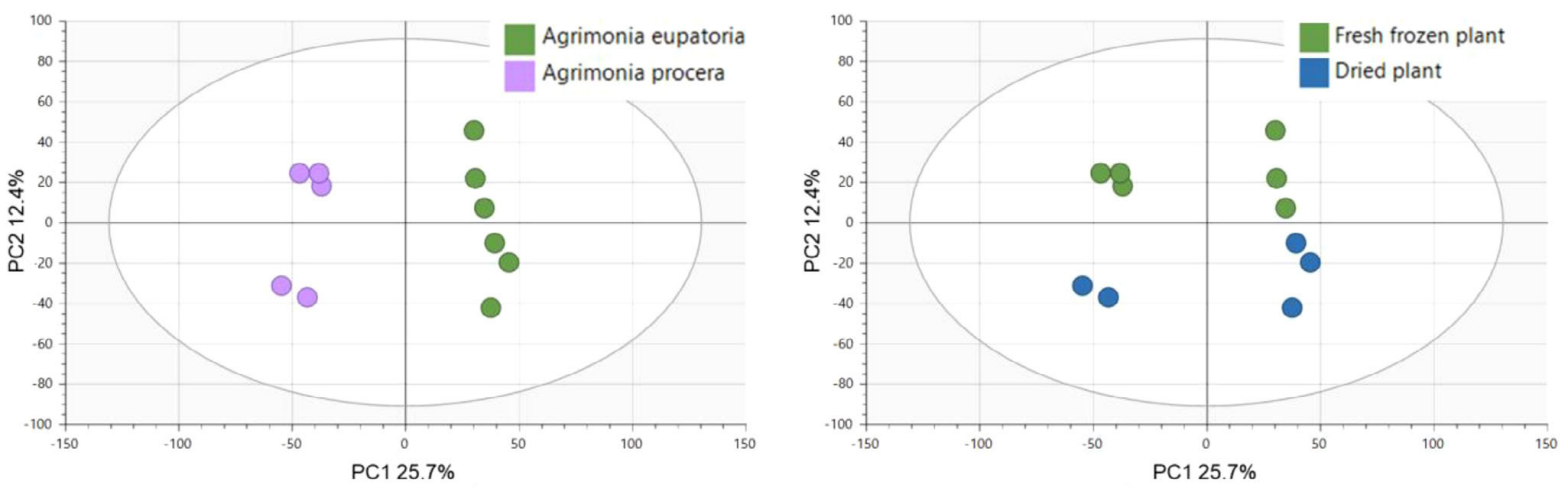
Principal component analysis (PCA) score plots (PC1 and PC2) of the agrimony roots clustered according to species and postharvest sample processing.

In line with expectation, the primary phytochemical variability was observed between the agrimony aerial parts and the roots, accounting for 13.7% of the variability on PC1 and 11% on PC2 (Figure S2). To visualize other potential factors impacting phytochemical profiles within these sample groups, subsequent PCA was performed on aerial parts and roots separately. The resulting score plots of only the aerial parts of the whole sample set identified species and postharvest sample processing as the primary influential factors, with species contributing 15.7% of the variability on PC1 and postharvest sample processing 7.9% on PC2 (Figure 1). Contrary to the distinct clustering of *A. eupatoria* against *A. procera* and the clearly clustered dried and fresh‐frozen samples, growth locality had no significant impact on the phytochemical profiles of agrimony aerial parts. The results obtained from PCA of root samples (Figure 2) were comparable to those obtained for the aerial parts.

**FIGURE 3:**
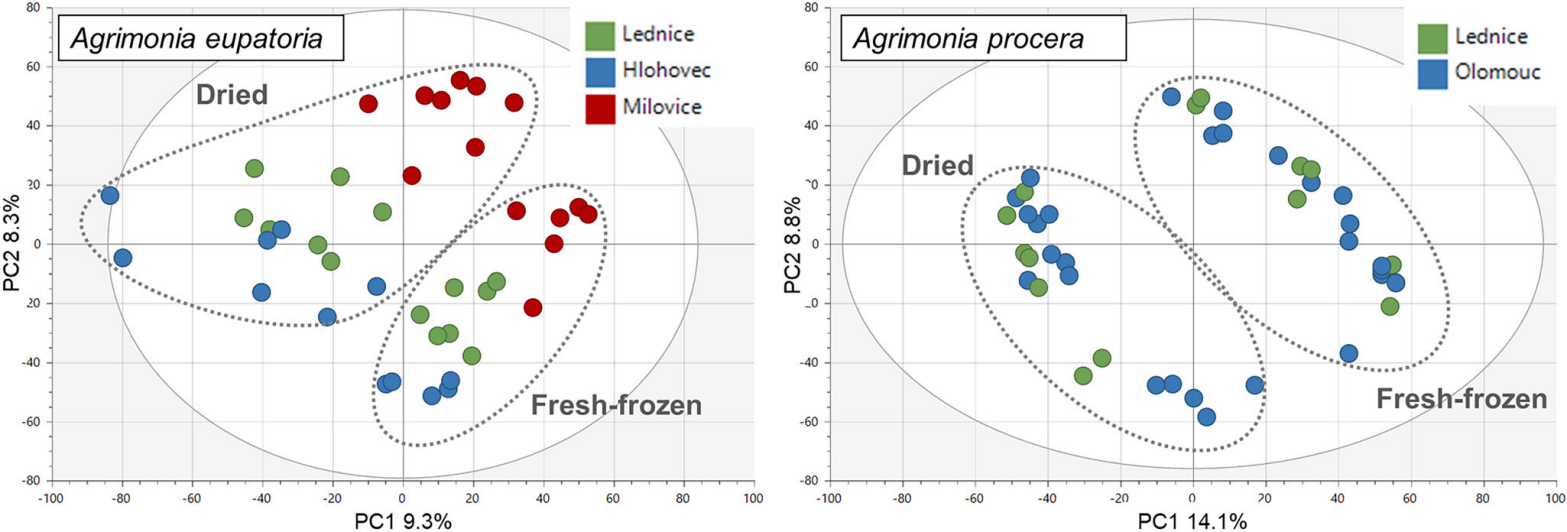
Principal component analysis (PCA) score plot (PC1 and PC2) of the aerial parts of agrimony samples clustered according to locality, with marked grouping of samples according to postharvest processing.

**FIGURE 4:**
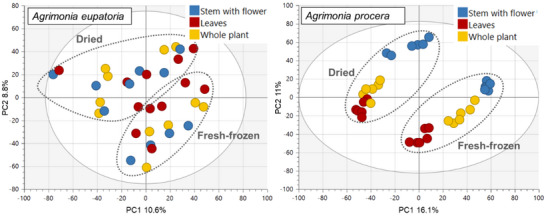
Principal component analysis (PCA) score plot (PC1 and PC2) of the aerial parts of agrimony samples clustered according to the plant part, with marked grouping of samples according to postharvest processing; only samples from the starting phase of flowering and full bloom were used for PCA as only these samples had plant parts collected separately.

PCA was also performed separately on data for each agrimony species, considering only the aerial plant parts (as the larger sample group compared to roots). In addition to growth locality and postharvest processing, the potential clustering of samples was also investigated on the basis of the type of aerial plant parts, such as leaves and stems with flowers. The aerial samples of *A. eupatoria* showed mild clustering according to the growth locality and postharvest processing, but no clustering according to the plant parts. In contrast, the *A. procera* samples were clearly divided on the basis of the postharvesting processing and plant parts, but not the growth locality (Figures 3 and 4).

Overall, the above‐described observations suggest that, in addition to the fundamental plant organs (roots and aerial parts), species and postharvest sample processing primarily influenced the agrimony phytochemical profile. The impact of locality and aerial plant part appeared to be minor but differed between the two tested agrimony species. *A. procera*, in the case of which the majority of samples were cultivated in the same locality, showed higher distinction by aerial plant parts than by locality, whereas *A. eupatoria*, being cultivated in three different locations, exhibited higher differentiation by the locality and no observable clustering by aerial plant parts. Thus, the comparably lower variability of *A. procera* samples, not influenced by locality, probably enabled distinguishing differences in phytochemical profiles of different aerial plant parts as well as enhanced the separation of dried and fresh‐frozen samples.

### 2.2 Polyphenol Profiles of Agrimony

From a total of 195 screened phenolic secondary metabolites, 62 were detected and tentatively identified across all agrimony extracts, with details provided in Table 1. Compound identification was based on HRMS/MS spectral matching with online databases (e.g., mzCloud, HMDB, and MassBank) or available literature and interpreted using the confidence level framework by Schymanski et al., where Level 1 indicates confirmation by analytical standard, and Level 5 represents tentative identification by exact mass only [15]. Due to the unavailability of analytical standards for such a high number of detected compounds, limitations in chromatographic resolution, and/or high similarity of their fragmentation spectra, several isomeric compounds could not be reliably distinguished.

**TABLE 1:** Polyphenolic compounds tentatively identified in agrimony extracts based on ultrahigh‐performance liquid chromatography and high‐resolution tandem mass spectrometry (UHPLC–HRMS/MS) analysis.

Compound name	Retention time (min)	Molecular formula	Measured *m/z* [M–H]^−^	Mass error (ppm)	HRMS/MS fragments (base peak in bold)	Identification level [15]
**1‐*O*‐Caffeoylquinic acid/4‐*O*‐Caffeoylquinic acid** ^a^	3.87	C_16_H_18_O_9_	353.0875	0.1	**135**, 173, 179, 191	3
**3‐Caffeoyl quinic acid**	3.73	C_16_H_18_O_9_	353.0875	0.5	85, **191**	2
**Afzelin**	6.88	C_21_H_20_O_10_	431.0985	−0.9	227, 255, 284, **285**	2
**Agrimoniin**	4.23	C_82_H_54_O_52_	934.0778^b^	1.6	**301**, 633, 783, 934, 935, 1085	2
**Apigenin**	7.86	C_15_H_10_O_5_	269.0463	0.3	107, **117**, 149, 151, 225	2
**Apigenin glucoside** ^a^	6.20	C_21_H_20_O_10_	431.0988	−0.3	**268**, 269	3
**Apigenin glucuronide/Baicalin** ^a^	6.03	C_21_H_18_O_11_	445.0786	−0.7	**269**	3
**Apigenin rutinoside** ^a^	6.10	C_27_H_30_O_14_	577.1575	−5.4	**269**	3
**Astragalin/Luteolin glucoside/Trifolin** ^a^	5.73	C_21_H_20_O_11_	447.0948	0.1	255, 271, 284, **285**	3
**Caffeic acid**	4.19	C_9_H_8_O_4_	179.0350	0.0	107, 134, **135**	2
**Caffeoylmalic acid**	4.20	C_13_H_12_O_8_	295.0459	−0.6	71, **115**, 133, 135, 179	2
**Catechin/Epicatechin** ^a^	3.49	C_15_H_14_O_6_	289.0719	1.4	**109**, 123, 125, 151, 203, 245	3
**Catechol**	2.80	C_6_H_6_O_2_	109.0296	2.8	**108**, 109	2
**Cirsimaritin**	8.39	C_17_H_14_O_6_	313.0718	0.5	255, **283**, 297, 298	2
**Coumaric acid isomer** ^a^	5.00	C_9_H_8_O_3_	163.0402	−0.8	93, **119**	3
**Coumaroylmalic acid**	4.80	C_13_H_12_O_7_	279.0510	0.6	115, **119**, 133, 163	2
**Diosmetin glucuronide** ^a^	6.14	C_22_H_20_O_12_	475.0885	−0.3	284, **299**	3
**Diosmin**	6.28	C_28_H_32_O_15_	607.1668	3.0	284, **299**	3
**Ellagic acid**	5.12	C_14_H_6_O_8_	300.9990	0.0	145, 173, 185, 201, 229, 283, 299	2
**Epigallocatechin/Gallocatechin** ^a^	3.70	C_15_H_14_O_7_	305.0668	−0.8	109, 121, **125**, 137, 161, 165	3
**Eriodictyol**	6.48	C_15_H_12_O_6_	287.0564	−1.3	**135**, 151,	2
**Eriodictyol glucoside** ^a^	5.15	C_21_H_22_O_11_	449.1095	−0.5	135, **151**, 287	3
**Ferulic acid/Isoferulic acid** ^a^	5.26	C_10_H_10_O_4_	193.0507	0.7	**133**, 134	3
**Feruloylquinic acid isomer** ^a^	6.63	C_17_H_20_O_9_	367.1034	−4.6	**93, 134, 173, 191, 193**	3
**Gallic acid**	1.88	C_7_H_6_O_5_	169.0143	3.4	79, 81, 124, **125**	2
**Gentisic acid/Protocatechuic acid/Pyrocatechuic acid** ^a^	3.15	C_7_H_6_O_4_	153.0194	1.5	**108**, 109	3
**Glucocaffeic acid**	3.82	C_15_H_18_O_9_	341.0875	−1.7	**135**, 179	3
**Hydroxybenzaldehyde/Benzoic acid** ^a^	4.14	C_7_H_6_O_2_	121.0295	−1.0	**92**, 121	3
**Hydroxybenzoic acid isomer/Salicylic acid** ^a^	3.52	C_7_H_6_O_3_	137.0244	1.1	65, **93**, 108	3
**Isoorientin/Orientin** ^a^	5.20	C_21_H_20_O_11_	447.0933	−1.0	285, 297, 298, **327**, 357	3
**Isoquercetin/Hyperoside/Quercetin galactoside/Quercetin glucoside/Spiraein** ^a^	5.85	C_21_H_20_O_12_	463.0892	0.5	255, 271, **300**, 301	3
**Isorhamnetin rutinoside** ^a^	6.25	C_28_H_32_O_16_	623.1617	2.3	299, 300, 314, 315	3
**Kaempferide/Isokaempferide** ^a^	7.96	C_16_H_12_O_6_	299.0564	0.0	**284**	3
**Kaempferol/Luteolin glucuronide** ^a^	5.55	C_21_H_18_O_12_	461.0735	−0.5	**285**	3
**Luteolin**	7.70	C_15_H_10_O_6_	285.0405	−0.4	**133**, 151, 187, 229	3
**Luteolin rutinoside/Nicotiflorin** ^a^	6.34	C_27_H_30_O_15_	593.1513	−0.4	284, **285**	3
**Luteolin/Kaempferol acetyl‐glucoside** ^a^ ^,^ ^c^	6.50	C_23_H_22_O_12_	489.1040	−1.9	255, **284**, 285	3
**Luteolin/Kaempferol malonyl‐glucoside** ^a^	6.48	C_24_H_22_O_14_	533.0937	−3.3	255, 284, **285**	3
**Methoxybenzoic acid**	6.45	C_8_H_8_O_3_	151.0400	−0.4	92, 106, **107**	2
**Methyl gallate**	3.69	C_8_H_8_O_5_	183.0299	‒0.7	**124**	2
***p*‐Coumaroyl quinic acid isomer** ^a^	3.49	C_16_H_18_O_8_	337.0927	0.2	**119, 163, 191**	3
**Peltatoside**	5.43	C_26_H_28_O_16_	595.1310	−1.8	**300**, 301	2
**Pentagalloylglucose**	5.04	C_41_H_32_O_26_	939.1149	−0.4	617, 769, 787	2
**Phloridzin**	6.05	C_21_H_24_O_10_	435.1296	−2.0	125, 167, 273	2
**Piceid**	5.99	C_20_H_22_O_8_	389.1238	−0.9	**227**	2
**Procyanidin type B** ^a^	3.04	C_30_H_26_O_12_	577.1354	−1.0	125, 245, 287, **289**, 407, 425	3
**Procyanidin type C** ^a^	3.08	C_45_H_38_O_18_	865.1995	0.3	125, 287, 289, 407, 425, 575, 577, 695	3
**Protocatechualdehyde**	3.38	C_7_H_6_O_3_	137.0245	1.4	92, **108**, 109, 136, 137	2
**Quercetin**	7.08	C_15_H_10_O_7_	301.0357	−4.6	107, 121, **151**, 178	2
**Quercetin acetyl‐glucoside** ^a^ ^,^ ^c^	5.98	C_23_H_22_O_13_	505.0985	−0.6	151, 178, 255, 271, **300**, 301, 463	3
**Quercetin diglucoside** ^a^	4.13	C_27_H_30_O_17_	625.1413	−0.6	299, 301, 462, 463	3
**Quercetin glucosyl‐rutinoside** ^a^	4.17	C_33_H_40_O_21_	771.2003	−0.8	299, 301, 462, 463, 609	3
**Quercetin malonyl‐glucoside** ^a^	5.98	C_24_H_22_O_15_	549.0896	−1.0	151, 178, 255, 271, **300**, 301, 463, 505	3
**Quercetin rhamnoside** ^a^	6.34	C_21_H_20_O_11_	447.0939	0.4	151, 178, 255, 271, **300**, 301	3
**Quercetin xyloside/Quercetin arabinoside** ^a^	6.07	C_20_H_18_O_11_	433.0778	−2.4	225, 255, 271, **300**, 301	3
**Quinic acid**	0.70	C_7_H_12_O_6_	191.0568	4.2	59, **85**, 87, 93, 108, 109, 127	2
**Rosmarinic acid**	5.93	C_18_H_16_O_8_	359.0772	3.3	133, 135, **161**, 179, 197	2
**Rutin**	5.83	C_27_H_30_O_16_	609.1480	−0.4	151, 178, 255, 271, **300**, 301,343	2
**Syringic acid**	4.45	C_9_H_10_O_5_	197.0455	1.6	95, **123**, 166, 182	2
**Taxifolin**	5.11	C_15_H_12_O_7_	303.0512	0.8	107, **125**, 151, 175, 177, 199, 217, 285	2
**Tiliroside**	7.29	C_30_H_26_O_13_	593.1306	−0.9	151, 255, **284**, 285, 447	2
**Vanillic acid**	4.17	C_8_H_8_O_4_	167.0350	0.5	65, 91, **108**, 152	2
**Vicenin 2**	4.63	C_27_H_30_O_15_	593.1517	−2.2	353, 383, 473, 503	2
**Vitexin/Isovitexin** ^a^	5.68	C_21_H_20_O_10_	431.0985	−0.7	283, **311**, 341	3

^a^ Alternative isomers structure.

^b^ [M–2H]^2−^

^c^ In source fragments of the malonyl glucoside counterpart (neutral loss of one CO_2_ molecule); that is, not an original compound.

Although many of the detected polyphenols (Table 1) have been previously reported in agrimony [3, 6, 8, 10], a number of others are, to the best of our knowledge, presented here for the first time. These include taxifolin (dihydroquercetin), feruloylquinic acid isomer, caffeoylmalic acid, apigenin rutinoside, catechol, cirsimaritin, diosmetin glucuronide, diosmin, eriodictyol, eriodictyol glucoside, isoorientin/orientin (luteolin 6‐C‐glucoside/luteolin 8‐C‐glucoside), isorhamnetin rutinoside, methyl gallate, methoxybenzoic acid, coumaroylmalic acid, pentagalloylglucose, phloridzin, hydroxybenzaldehyde/benzoic acid, piceid (resveratrol 3‐*O*‐glucoside), quercetin diglucoside, quercetin glucosyl‐rutinoside, quercetin xyloside/quercetin arabinoside, syringic acid, and vicenin 2 (apigenin 6,8‐di‐C‐glucoside).

The possible impact of specific cultivation and/or processing factors on the polyphenols profile of agrimony was evaluated on the basis of the occurrence of the detected compounds in the tested extracts. Although some compounds were consistently found across all samples (taxifolin, feruloylquinic acid isomer, quercetin xyloside/arabinoside, catechol, eriodictyol, pentagalloylglucose, and quercetin diglucoside), others, such as phloridzin and quercetin glucosyl‐rutinoside, were detected only in certain samples, either specific to a particular species or locality. The occurrence of some other compounds (e.g., methyl gallate and methoxybenzoic acid) appeared rather random, without any observable trend. Interestingly, certain compounds such as diosmin and cirsimaritin were detected only in samples from the vegetative phase of a “wild” *A. eupatoria* growing naturally in the Milovice locality (the latter compound only in the fresh plant). Syringic acid was also detected in the same plant, but exclusively in the root samples.

Considering the general differences in the phytochemical profiles of the fundamental plant structures, as supported by our PCA results, agrimony roots and aerial parts were further evaluated separately [14, 16].

#### 2.2.1 Roots

In line with our results of the total phytochemical profile, significant differences between agrimony root and aerial samples were also confirmed for both the qualitative (occurrence) and quantitative (signal intensity) profiles of polyphenols. Although the chromatographic records of root samples exhibited considerable richness, only a limited number of the screened compounds, 35 in total, were identified across all root samples. This is most likely due to the primary focus of this study, and thus also of the compiled “in‐house” database, on polyphenols, whereas roots are generally rich in other types of compounds such as triterpenoids [17, 18]. In addition, polyphenols in roots might be bound to structural polysaccharides (e.g., cellulose), which would limit their extractability and thus detectability [19, 20].

Flavonoids and tannins were the primary compounds detected in the roots, accounting for 58%–75% of the identified polyphenols. However, the content of individual flavonoids was generally lower compared to the aerial parts. Phenolic acids, when present, also occurred in significantly lower amounts, representing a maximum of 6% of all identified polyphenols in the roots, in contrast to 12%–35% in the aerial parts. These findings are consistent with those of Dos Santos Szewczyk et al., who reported reduced total phenolic, flavonoid, and phenolic acid contents in the roots of *Alchemilla acutiloba* (a member of the Rosaceae family, like agrimony) compared to its aerial parts, along with reduced levels of individual flavonoids [21]. To date, no study has systematically investigated phenolic compounds in agrimony roots.

Although the overall content of the detected polyphenols was typically higher in the agrimony aerial parts, some compounds were present in significantly greater quantities (*p* < 0.05) in the roots. These included agrimoniin, catechin/epicatechin, B‐type procyanidins, gallic acid, and pentagalloylglucose. Hoffmann et al. reported that the roots of certain Rosaceae species (strawberry, rose, pear, plum) serve as sites of flavonoid biosynthesis, particularly of flavan‐3‐ols such as catechin [22]. Similarly, Enayati et al., who examined differences between aerial parts and roots of *Potentilla reptans* L. from the Rosaceae family, identified flavan‐3‐ols as the predominant compounds in plant roots [23]. As agrimony also belongs to the Rosaceae family, our findings of elevated levels of these compounds in its roots are consistent with these previous reports.

#### 2.2.2 Aerial Parts

##### 2.2.2.1 Impact of Species

The polyphenol profiles of agrimony aerial parts showed clear species‐dependent variability, both in terms of compound occurrence and detected levels. The content (based on signal intensity) of gentisic acid/protocatechuic acid/pyrocatechuic acid, quercetin malonyl‐glucoside, quercetin xyloside/quercetin arabinoside, and vicenin 2 was significantly higher (*p* < 0.05) in extracts prepared from *A. eupatoria* compared to *A. procera*.

Some secondary metabolites were detected exclusively in *A. eupatoria*, namely, phloridzin, the luteolin C‐glycosides isoorientin/orientin, and the apigenin C‐glycosides isovitexin/vitexin. In agreement with our findings, isovitexin and vitexin were previously reported by Granica et al. as possible chemotaxonomic markers for differentiating *A. eupatoria* and *A. procera* [6]. To the best of our knowledge, phloridzin and isoorientin/orientin have not yet been reported to occur in agrimony; nevertheless, they are typical of a number of other plants. Orientin has been detected in species such as *Fagopyrum esculentum*, and phloridzin in several medicinal herbs, including *Salvia officinalis* L., *Alchemilla vulgaris*, and *Thymus serpyllum* L. [16, 24, 25]. The occurrence data obtained for these compounds indicate their potential use as markers for distinguishing *A. eupatoria* and *A. procera*, alongside vitexin/isovitexin.

In line with other studies, we also observed a significantly higher (*p* < 0.05) content of agrimoniin and apigenin glucoside in *A. procera* compared to *A. eupatoria* [6, 10]. One specific apigenin glucoside isomer, apigenin 7‐*O*‐glycoside, was reported by Granica et al. as a possible marker for *A. procera* [6]. However, as we were not able to identify the specific position of the glycosidic bond in the apigenin glucoside isomer, we could neither confirm nor contradict their hypothesis. Although significantly elevated levels were found in our *A. procera* samples, the non‐specified apigenin glucoside was also detected to some extent in *A. eupatoria*, suggesting either that apigenin 7‐*O*‐glucoside might not be exclusive to *A. procera*, or that a different apigenin glucoside isomer was detected in our samples. Another compound found exclusively in *A. procera* was apigenin rutinoside. However, as it was detected mainly in samples from the vegetative and early flowering phases and only in four samples from full bloom, it appears to be an unsuitable marker of this species.

##### 2.2.2.2 Impact of Postharvest Processing

Postharvest processing of agrimony aerial parts had a noticeable impact on their polyphenol content across all samples. Consistent with expectations, higher polyphenol levels were observed in fresh‐frozen samples compared to those dried at 40°C. This corroborates the findings of Dziadek et al., who reported a decline in total polyphenol content in *Salvia* species dried at temperatures exceeding 30°C [26]. Similarly, Rababah et al. found higher concentrations of total phenolics and flavonoids in fresh sage, mint, lemon balm, and thyme compared to their oven‐dried (40°C) counterparts [27]. The rate of polyphenol loss during air drying varied among individual compounds and could probably be caused by oxidation, mainly by the activity of polyphenol oxidases. These enzymes represent a diverse group with different optimal parameters depending on the substrate. Their optimal activity temperature ranges approximately from 10°C to 65°C; therefore, it is plausible that agrimony drying at 40°C increased the activity of certain polyphenol oxidases, resulting in a reduced polyphenol content [28, 29, 30].

##### 2.2.2.3 Impact of Growth Locality

Polyphenolic profiles were compared across all agrimony samples with regard to their geographic origin. The most pronounced differences, both in qualitative and quantitative representation of polyphenols, were observed in *A. eupatoria* cultivated at the Lednice locality. The content of apigenin was significantly lower (*p* < 0.05) compared to all samples from other localities. Moreover, several bioactive secondary metabolites commonly reported in agrimony (rutin, apigenin glucuronide/baicalin, kaempferol glucuronide/luteolin glucuronide, and luteolin rutinoside/nicotiflorin) [3, 6, 8, 10] as well as some compounds identified in agrimony for the first time in this study (isorhamnetin rutinoside and quercetin glucosyl‐rutinoside), were not detected in *A. eupatoria* from Lednice. Conversely, the content of afzelin (kaempferol glucoside) in *A. eupatoria* grown in Lednice was significantly higher (*p* < 0.05) compared to all other samples. Interestingly, *A. procera* cultivated at the same site under identical environmental conditions did not exhibit any of these deviations. This may indicate a higher sensitivity of *A. eupatoria* to lower intensity fertilization or nutrient deficiency compared to *A. procera*.

Another notable feature of the *A. eupatoria* samples from this locality was the presence of coumaroylmalic acid and ellagic acid, the latter of which could be released from ellagitannins. This transformation may be driven by various factors such as light, high concentrations of acids or bases, or enzymatic activity [10]. However, we are unable to determine the specific factor responsible in this case.

The aforementioned differences in the polyphenolic profile of *A. eupatoria* from controlled cultivation in Lednice were highly unexpected, as the highest variability was initially anticipated in the wild‐grown plants from Milovice site. However, those samples displayed only minor deviation from the others, such as the absence of isorhamnetin glucoside, afzelin, and eriodictyol glucoside.

##### 2.2.2.4 Impact of Aerial Plant Parts

The agrimony aerial parts investigated in this study included the apex, leaves, stem with flowers, and the whole flower. The highest content of polyphenolic compounds was most frequently detected in the leaves, regardless of the postharvest processing method. However, in *A. eupatoria* from Lednice locality, several tannin compounds (viz., epigallocatechin/gallocatechin and ellagic acid) were present in significantly higher (*p* < 0.05) amounts in stems compared to other aerial plant parts. Therefore, the quantitative polyphenolic profile of the whole plant may vary depending on the ratio of stems to leaves, which aligns with the findings of Karlińska et al., who concluded that polyphenol profiles in commercial herbal products depend on the stem‐to‐leaf ratio used in the preparation, as compound quantities, and presumably biological effects, differ between these plant parts [10]. Our results confirm that this applies not only to dried herbal products but also to fresh herb material.

##### 2.2.2.5 Impact of Ontogenetic Phase

**FIGURE 5:**
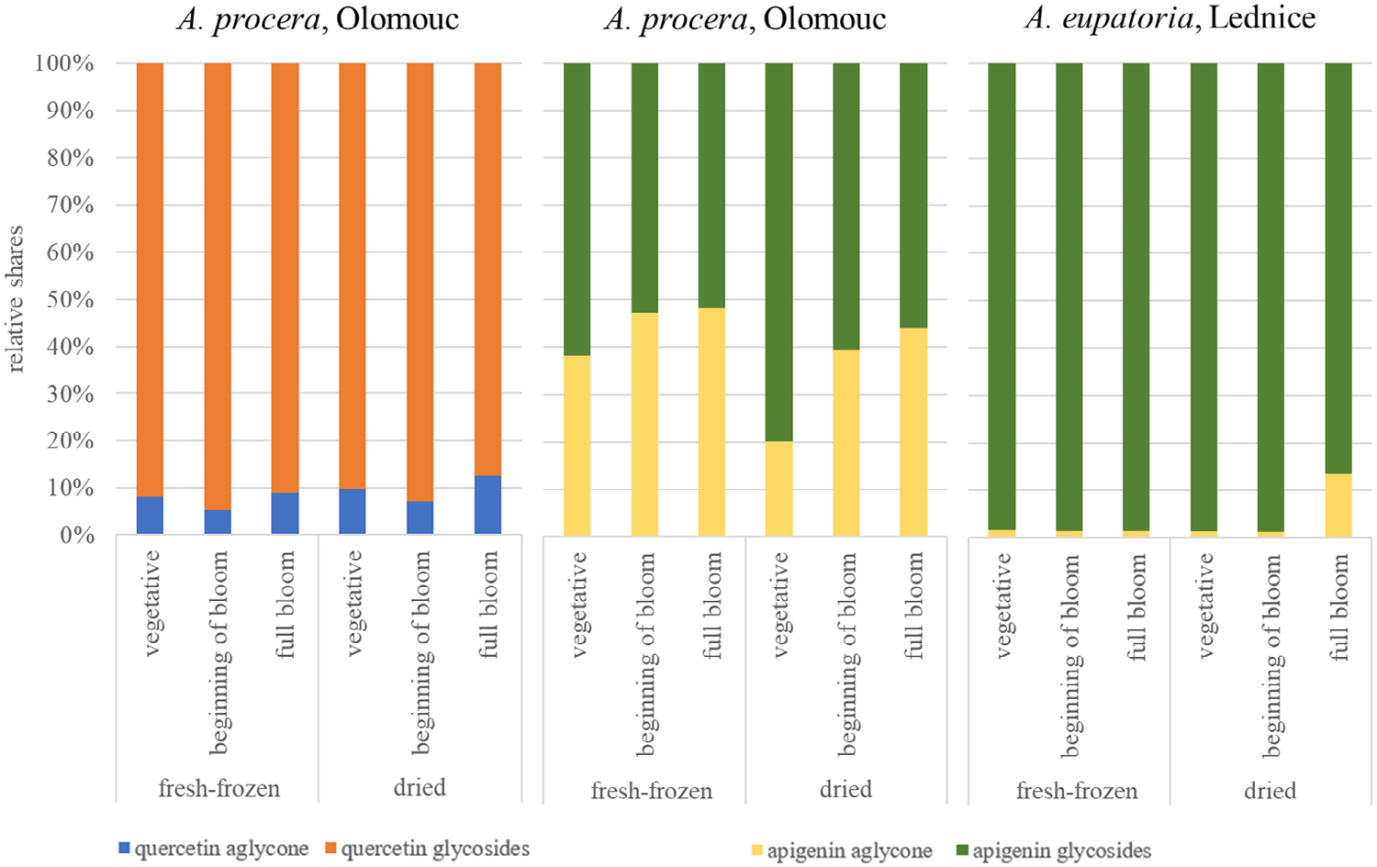
Relative abundance of quercetin and apigenin aglycones and glycosides in selected agrimony samples (aerial parts) across different ontogenetic phases.

Analysis of samples from all aerial parts revealed relatively stable qualitative polyphenol profiles throughout ontogenesis; however, notable differences in the levels of specific compounds were observed between the vegetative phase, beginning of flowering, and full bloom phases (the senescence phase was not included, as only root samples were collected at this stage). Diosmetin glucuronide, caffeic acid, and luteolin/kaempferol malonyl‐glucoside exhibited significantly higher (*p* < 0.05) levels during the vegetative phase across all samples. The differences in the levels of these compounds between the vegetative and the two subsequent phases might have been, however, strongly influenced by the higher proportion of leaves to stems in the samples from the vegetative phase (as the plants are at the early stage of growth), with this ratio later shifting as the stems develop further. Generalić et al. conducted a comparative assessment of variations in phenolic compounds during the growth of *S. officinalis* L. leaves, indicating that environmental factors strongly influence polyphenol content in plants, leading to considerable fluctuations [13]. This observation in other plants is supported by our results. However, our study aimed to elucidate trends in the polyphenol profiles during ontogenesis in a greater detail. We focused on changes in the relative representation of flavonoid derivatives throughout plant growth, particularly the relative shares of aglycones and glycosides of apigenin and quercetin. Kaempferol and luteolin were not included, as it was not possible to distinguish their glycosides from each other in this study. The percentage of each identified flavonoid aglycone and its glycosides was calculated for the samples of whole plant collected at each phase, enabling comparison of their relative ratios. The highest relative share of glycosides was observed for quercetin, usually at the beginning of the bloom phase, whereas the lowest ratio varied between the vegetative phase and full bloom. For apigenin, the highest ratio of glycosides in *A. procera* was mainly in the vegetative phase, with a decreasing trend in the subsequent phases. In *A. eupatoria*, the trend was similar to that of quercetin, with the highest ratio of glycosides at the beginning of the bloom phase, with the exception of samples from Lednice. No significant differences in these trends were observed between dried and fresh‐frozen herbs. Quercetin exhibited a predominance of glycosides (typically more than 80%) and minimal aglycone content, whereas apigenin displayed more balanced ratios, with aglycone shares reaching up to almost 50% in some cases. An exception was noted in samples of *A. eupatoria* from the Lednice locality, where the relative share of apigenin aglycone was notably low (not exceeding 15%), likely attributable to its reduced apigenin content compared to other samples. These results are illustrated in Figure 5.

### 2.3 Antimicrobial Potential of Agrimony and Correlation with Detected Polyphenols

Significant variations in efficacy against the tested microorganisms were observed among the agrimony samples. Although no notable antimicrobial activity was detected against anaerobic bacteria *Cutibacterium acnes* in any extract, IC_50_ values were measurable for other microorganisms, including the Gram‐negative (G−) *Pseudomonas aeruginosa*, Gram‐positive (G+) *Staphylococcus aureus*, methicillin‐resistant *S. aureus* (MRSA)*, Enterococcus faecalis*, vancomycin‐resistant *Enterococcus* sp. (VRE), and the yeast *Candida albicans*. These findings are consistent with previous reports indicating the effectiveness of various agrimony extracts against some of these pathogens [3, 31]. The detailed overview of the detected antimicrobial activity is provided in Tables S1–S6.

In general, the agrimony extracts exhibited stronger inhibitory effects against G+ bacteria than G− bacteria, in agreement with the findings of Muruzović et al. [4]. The higher resistance of G− bacteria to plant‐derived antimicrobials is commonly attributed to their outer membrane structure, which limits the permeability of active compounds [32]. Notably, root extracts demonstrated the highest antimicrobial activity in most cases across all tested samples. This may be partially explained by their higher dry matter content compared to aerial plant parts, resulting in elevated levels of extracted bioactive compounds. Nevertheless, the differences were substantial, corroborating the findings of Bělonožníková et al., who also reported the highest antimicrobial activity against *P. aeruginosa* in roots when comparing different plant parts of *A. eupatoria* [33].

The effects of species, postharvest processing, growth locality, plant part, and ontogenetic phase on antimicrobial activity were statistically assessed using one‐way analysis of variance (ANOVA) or two‐sample *t*‐test assuming equal variance. As no significant antimicrobial effect was observed against *C. acnes*, this bacterium was excluded from further statistical analyses.

The impact of individual factors varied depending on the target microorganism. According to ANOVA and *t*‐test results, species significantly (*p* < 0.05) influenced antimicrobial activity mainly against G+ bacteria but had no significant effect on activity against *P. aeruginosa* and *C. albicans*.

Postharvest processing significantly (*p* < 0.05) affected activity against *S. aureus* and *E. faecalis*. For activity against *P. aeruginosa* and MRSA, significant (*p* < 0.05) influence was observed only in the case of *A. procera*, whereas no impact was noted on activity against VRE and *C. albicans*.

Growth locality significantly (*p* < 0.05) influenced antimicrobial activity only against *S. aureus* and VRE, whereas activity against MRSA was impacted only for *A. eupatoria*. No significant locality‐related effects were observed for other microorganisms.

The observed effects of agrimony plant part and ontogenesis were largely driven by the root samples, which, as already mentioned, exhibited significantly different antimicrobial activities compared to aerial parts. In many cases, statistically significant (*p* < 0.05) differences disappeared when root samples were excluded from the analysis. Without roots, plant parts significantly (*p* < 0.05) influenced activity only against MRSA (and to a lesser extent against *S. aureus* in the case of *A. procera*), whereas no impact was observed for the remaining organisms. Similarly, ontogenetic phase did not significantly affect antimicrobial activity once roots were excluded. An exception was observed for *C. albicans*, where fresh‐frozen agrimony samples from the vegetative phase exhibited higher activity than all the other samples.

Overall, these results demonstrate that all investigated factors influence agrimony's antimicrobial activity against bacteria and yeast. Although species and postharvest processing significantly affected antimicrobial activity against almost all tested microorganisms, other factors, such as particularly ontogenetic phase, had only a minor impact on activity against specific microorganism. This finding aligns with the PCA results, which showed no clustering based on agrimony developmental stage (considering the aerial plant parts only). Generalić et al. also reported changes in antibacterial activity against two G+ (*Bacillus cereus* and *S. aureus*) and two G− (*Escherichia coli* and *Salmonella infantis*) bacteria in *S. officinalis* L. (Rosaceae family) during different ontogenetic phases [13]. However, they did not statistically evaluate the significance of these differences using ANOVA or similar tests. The higher activity reported against *S. aureus* compared to our results on agrimony samples may reflect species‐specific differences in polyphenolic composition of the two plants. To our knowledge, no prior study has systematically explored the impact of environmental and processing factors on the antimicrobial activity of agrimony extracts in such detail.

Furthermore, the association between antimicrobial activity and specific detected polyphenols was investigated by calculating Pearson's correlation coefficients between the measured IC_50_ values (Tables S1–S6) and the peak areas of the compounds. When evaluating Pearson's correlation coefficients, we used the classification described by Care et al., where coefficient values above 0.6 (or below −0.6) are considered strong correlations, and values between 0.4 and 0.59 (or −0.4 and −0.59) are considered moderate [34]. As IC_50_ values are inversely related to biological activity (i.e., the lower the IC_50_, the higher the observed activity), negative coefficients represent positive correlations in this study. Four polyphenolic compounds, namely, agrimoniin, pentagalloylglucose, gallic acid, and phloridzin, met criteria for moderate or strong correlation (Table 2).

**TABLE 2:** Pearson's correlation coefficients indicating moderate to strong correlations between antimicrobial activities of agrimony extracts and levels of detected bioactive compounds.

Compound	Pearson's correlation coefficient					
	*Staphylococcus aureus*	*Enterococcus faecalis*	MRSA	VRE	*Pseudomonas aeruginosa*	*Candida albicans*
Agrimoniin	−0.57	—	—	—	−0.63	—
Gallic acid	−0.53	—	—	—	—	—
Pentagalloylglucose	−0.50	—	—	—	−0.52	—
Phloridzin	—	—	—	−0.50	—	—

Abbreviations: MRSA, methicillin‐resistant *Staphylococcus aureus;* VRE, vancomycin‐resistant *Enterococcus* sp.

Gallic acid has been documented to exhibit antibacterial and antifungal properties [35, 36, 37]. Similarly, pentagalloylglucose and phloridzin have been reported to possess antimicrobial activity [38, 39]. Although agrimoniin has been suggested to contribute to certain beneficial properties of agrimony extract, it has not, to the best of our knowledge, been reported to display antimicrobial, particularly antibacterial, activity [6]. These findings indicate that among the detected and identified polyphenolic compounds, agrimoniin, gallic acid, pentagalloylglucose, and phloridzin may primarily contribute to the antibacterial activity of agrimony against both G+ and G− bacteria. However, to confirm the role of these correlated compounds as the primary antibacterial agents in the extracts, further research should be carried out.

A study with a similar objective, aiming to identify the compounds responsible for the antibacterial activity of agrimony extracts against *S. aureus*, was conducted by Ginovyan et al. [40]. Using the TLC‐bioautography technique, they concluded that the observed activity is likely attributable to more than one compound, specifically to interactions of many present compounds. Similar conclusions were drawn by Bělonožníková et al., who suggested that antimicrobial activity depends not on a single substance, but rather on the combined effect of multiple secondary metabolites [33]. These conclusions might partially explain results of our study, where no significant correlation was found between antimicrobial activity and any individual detected compound for half of the tested microorganisms (viz., *E. faecalis*, MRSA, and *C. albicans)*, despite the extracts showing strong activity against them.

## 3 Conclusions

*A. eupatoria* L. and, to a lesser extent, *A. procera* WALLR., have been used in traditional medicine for centuries. However, the effectiveness of their non‐standardized preparations might be rather inconsistent, due in part to variability in the phytochemical composition driven by various cultivation and processing factors. This study provides a comprehensive evaluation of how species, growth locality, plant parts, ontogenetic stage, and postharvest processing affect the phytochemical profile and antimicrobial activity of agrimony extracts.

Using UHPLC–HRMS/MS combined with multivariate statistical analysis, we demonstrated that agrimony phytochemistry is strongly shaped by species identity and postharvest processing, with smaller contributions from growth locality, ontogenetic stage, and plant part (apart from the expected fundamental differences between the roots and aerial parts). Targeted screening of polyphenols (as major bioactive secondary metabolites of agrimony) identified several compounds that, to the best of our knowledge, are reported in agrimony for the first time in this study. We proposed phloridzin and luteolin C‐glycosides (isoorientin/orientin) as potential chemotaxonomic markers for *A. eupatoria*, expanding the currently known marker panel. Slight variation in polyphenol profiles among agrimony samples from different growing localities was observed. Regardless of species and locality, leaves consistently showed the highest polyphenol content, whereas roots, despite lower compound diversity, exhibited significantly elevated levels of catechins and procyanidins. Ontogenetic trends indicated highest polyphenol content in the vegetative phase, with distinct patterns in the ratio of flavonoid glycosides to aglycones throughout plant development. Drying at 40°C was shown to reduce polyphenol levels, possibly due to enzyme‐mediated oxidation processes.

Antimicrobial activity was particularly high against Gram‐positive bacteria and was closely associated with root extracts. As expected, the differences in observed activity across all the agrimony extracts were closely associated with variability in the detected phytochemical profiles, with species and postharvest processing being the primary influencing factors. Extracts of fresh‐frozen *A. eupatoria* showed high efficiency against *Enterococcus* bacteria (*E. faecalis*, VRE), whereas *A. procera* extracts were most effective against *S. aureus* and MRSA. Good inhibition was also shown for the yeast *C. albicans*. Among the 62 polyphenols detected and tentatively identified across all the agrimony extracts, agrimoniin, gallic acid, pentagalloylglucose, and phloridzin showed moderate to strong correlations with observed antibacterial activity, although no single compound explained the activity across all tested microorganisms. Our results support the hypothesis that synergistic effects among multiple bioactive compounds play a key role in the antimicrobial properties of agrimony extracts.

Together, these findings highlight the considerable variability in agrimony's phytochemistry and bioactivity associated with healing effect and stress the importance of standardizing plant material and processing methods to ensure the consistent quality and efficacy of herbal medicinal products. Future work should focus on isolating and characterizing the individual and synergistic contributions of key bioactive compounds, as well as evaluating other pharmacological properties beyond antimicrobial activity.

## 4 Experimental Section

### 4.1 Chemicals

Ammonium formate (purity >99.9%) and ethanol (p.a.) were supplied by Merck (Germany). HPLC methanol (MeOH, purity >99.9%) and formic acid (FA, purity >99.9%) were purchased from Honeywell Riedel‐de Haën (Germany). Deionized water was obtained from an internal Milli‐Q system (Merck, Germany). For antimicrobial testing, MH broth (Mueller Hinton Broth; Merck, Germany) was used.

### 4.2 Plant Samples

Two agrimony species (*A. eupatoria* L. and *A. procera* WALLR.) were provided by the project partner Mendel University in Brno, Faculty of Horticulture, Department of Growing Vegetable and Floriculture, and were authenticated on the basis of morphological features by Jarmila Neugebauerová according to literature [41, 42, 43]. The plant material of *A. eupatoria* was also authenticated according to the European part of the Czech Pharmacopoeia [44]. The identification of plants was verified by Radomír Řepka. Vouchers are stored at Mendel University Brno, Faculty of Horticulture. The list of samples (listed as S1–S6), place of collection, inventory, and herbarium code is given in Table S7, together with the photo documentation of the herbarium item of wild *A. eupatoria* collected in the locality of Milovice (sample S4; Figure S3). The herbarium items are stored in the depository of the Regionální muzeum Mikulov (Regional Museum in Mikulov). Although samples S5 and S6 do not have a herbarium accession, they are part of the genetic resources collection, and their seeds are available in the gene bank under the codes 09A0500001 and 09A0500002 [45, 46]. Sample S5 was initially assumed to represent the *A. eupatoria* species. However, UHPLC–HRMS/MS analysis and statistical evaluation conducted in this study clearly revealed that the phytochemical profile of S5‐related plant samples corresponds to *A. procera*. Consequently, the authors collectively decided to further evaluate the plant S5 as *A. procera*. This finding underscores the importance of phytochemical analysis in botany and traditional medicine for accurate plant species identification, particularly in cases involving species with high morphological similarity, such as the *A. eupatoria* and *A. procera* examined in this study.

The plants were collected at four points through their ontogenesis (in May, June, July, and October) at four different localities; in three of them (Lednice: 48.7947658 N, 16.7985383 E; Hlohovec: 48.7630025 N, 16.7600808 E; Olomouc: 49.5715003 N, 17.2815117 E), the agrimony was grown under controlled conditions, whereas in the fourth locality (Milovice: 48.8489458 N, 16.6918897 E), samples were collected from nature. In May, plant in the vegetative state was collected as all the aerial parts together (as in this phase the plant consists mainly of leaves and a little bit of stem), and in June and July, besides whole plant, leaves and stems with flowers were also collected separately. In October, that is, in the senescence period, only roots (with rhizomes) were collected. All the samples are listed in Table S8. After collection, part of fresh samples was cryogenically homogenized by liquid nitrogen to avoid any changes of bioactive metabolites, and then, the homogenized plant materials were stored in freezer (−18°C), and the other part of these samples was dried in a laboratory oven with circulating hot air (24‐h cycles, 40°C) and afterwards homogenized to get fine powder. The dry matter content of the fresh‐frozen samples was measured using an HG63 halogen moisture analyzer (Mettler Toledo, Germany), and all data obtained in this study were recalculated accordingly.

### 4.3 Preparation of Plant Extracts

Sample extraction was performed according to the procedures used in a folk medicine. A tincture was chosen for the purpose of this study as higher antimicrobial activity has been reported for this type of extract compared to the aqueous one [4]. The extraction procedure was chosen on the basis of publicly accessible sources, including internet websites and herbal books recommended on various platforms for homemade extracts preparation [47]. To follow recommended set‐up (one part of herb to five parts of extraction solvent), 4 g of each type of plant material was mixed with 20 mL of 40% (v/v) ethanol, with few exceptions (see Supporting Information section, Table S8), where the ratio had to be changed due to insufficient wettability. The suspensions were left for 7 days in the dark at 23°C, shaken at least once a day. After 7 days, the liquid phase was filtered using filter paper (84 g/m^2^) and subsequently passed through microfilters (0.2 µm PVDF, Chromservis).

### 4.4 UHPLC–HRMS/MS Analysis

For the plant extracts analysis, UHPLC–HRMS/MS technique was employed using a Dionex UltiMate 3000 chromatograph (Thermo Fisher Scientific, USA) coupled with a TripleTOF 6600 mass spectrometer (SCIEX, Canada) with Q‐TOF mass analyzer. Chromatographic separation was carried out on Acquity HSS T3 (2.1 × 100 mm^2^, 1.8 µm, Waters) analytical column held at 45°C. The mobile phase consisted of A: deionized water with 5 mM ammonium formate and 0.1% formic acid and B: methanol with 5 mM ammonium formate and 0.1% formic acid. The gradient was used as follows: initial (5% B), 0–5 min (5%–50% B), 5–11 min (50%–100% B), 11–18 min (100% B), and 18–20 min (5% B). The injection volume was 2 µL, and the flow rate was 0.4 mL/min. Samples were analyzed separately in positive and negative modes of electrospray ionization (ESI) under following conditions: capillary temperature 480°C, capillary voltage −4500/+5000 V, and collision energy 35 V with an energy spread of ±15 V. For the acquisition of MS and MS/MS data, Full scan and Information Dependent Acquisition (IDA) methods were used. Although full MS data were recorded for *m/z* 100–1200, product ion MS/MS data were collected for *m/z* 50–1200. After every 10 samples, automatic *m/z* calibration was performed using positive or negative ESI calibration solution (SCIEX, Canada). The quality control (QC) samples and blank samples were analyzed within the sequence to monitor potential drift of retention times and/or changes in detector response together with potential carry‐over effects (Figure S1). QC samples were prepared by pooling a few microliters of each sample extract. Blank sample consisted of pure extraction solvent only.

### 4.5 Targeted Screening and Identification of Polyphenols

To perform the targeted screening of polyphenols in the plant extracts measured by HPLC–HRMS/MS, a unique database of compounds of interest was created. The existing “in‐house” library, containing information on nearly 600 bioactive secondary metabolites (mainly polyphenols) reported in scientific literature to occur in common medicinal plants, was modified and extended with compounds listed in studies focused on the phytochemical profiles of various agrimony species. In the next phase, compounds having MS/MS spectrum available in online spectral libraries (www.mzcloud.org, www.pubchem.com, www.massbank.eu, metlincloud2.massconsortium.com) were chosen to reduce the number of screened compounds. Overall, 195 bioactive compounds were included in the final database, which consists of the following available information: (i) alternative common names, (ii) molecular formula, (iii) chemical class, (iv) chemical identifiers (CAS), and (v) online spectral libraries containing the available MS/MS spectra. For data processing, the SCIEX OS software (version 1.5.0.23389, SCIEX, Canada) was used, and the criteria for compound detection and identification were as follows: (i) measured exact mass on 4 decimal places, (ii) mass error (<5 ppm), (iii) ion intensity threshold above 1000, and (iv) conformity of mass fragmentation spectra with those in online spectral libraries.

### 4.6 Antimicrobial Activity Testing

Antimicrobial activity was tested using broth microdilution method according to ISO standard 20776‐1 [31, 48]. Tested microorganism included *S. aureus* (ATCC 25923), *P. aeruginosa* (CCM 3855), *C. albicans* (DBM 2164), *E. faecalis* (CCM 4224), *C. acnes* (CCM 7417), MRSA (NEM 449 [49]), and VRE (NEM 2159). Both resistant strains were isolated and identified in the Laboratory of Medical Microbiology, Česká Laboratorní, s.r.o. (Prague, Czech Republic). All samples tested ranged from 0.002 to 1 g/L in triplicates. The activity was evaluated as IC_50_ (half maximal concentration; the concentration of sample and antibiotic that kills exactly 50% of the bacterial population) after 24 h of incubation (37°C, 120 rpm/min) using the web calculator https://www.aatbio.com/tools/ic50‐calculator/.

### 4.7 Statistical Analysis of Generated Data

The processing of raw UHPLC–MS/MS data prior to statistical analysis was performed with MarkerView software 1.3 (SCIEX, Canada). For non‐targeted peak detection, the parameters were set as follows: minimum peak width 0.02 Da, noise threshold 10, subtraction multiple factor 1.5. Peak alignment parameters were set to mass tolerance 0.01 Da and retention time tolerance 0.1 min. After peak detection and alignment, total area sum normalization was performed to obtain the total phytochemical profile of each sample extract. Further chemometrics evaluation was performed in SIMCA 17.0 software (Umetrics, Sweden; https://umetrics.com/). For creating PCA models, logarithmic transformation and Pareto scaling were set.

ANOVA and two‐sample *t*‐test assuming equal variance (performed using Microsoft Office Excel function Data Analysis) were used on the data obtained by targeted screening to determine differences between groups and correlations to determine the relationship between antimicrobial activity and identified polyphenols. For both ANOVA and the *t*‐test, the measured antimicrobial activity of the samples against each bacterium was separated into groups according to the observed factor, and statistical analysis was then performed to determine differences between these groups.

## Author Contributions

Anna Loučková performed all the experiments connected to the analytical part of extract testing, collected and analyzed all the data, and wrote the first draft of the manuscript. Jarmila Neugebauerová provided and identified plant material, provided supervision connected to the botanical part of the manuscript, and modified the draft of the manuscript. Bára Křížkovská did the experiments connected to the antimicrobial activity. Marie Zlechovcová participated in the design of the study and reviewed and modified the manuscript draft. Kateřina Šebelová helped with the data analysis. Jan Lipov supervised the whole process of antimicrobial activity testing and reviewed the manuscript. Jitka Viktorová participated in the design of the study, helped with the experiments connected to the antimicrobial activity, reviewed and modified the manuscript draft. Jana Hajšlová designed the study, supervised the whole process, and reviewed and modified the manuscript draft. All authors read and approved the final manuscript.

## Conflicts of Interest

The authors declare no conflicts of interest.

## Supporting information




**Supporting file 1**: Supporting Information.docx

## Data Availability

The data that support the findings of this study are available from the corresponding author upon reasonable request.
