# Supplementary material for: Impact of Species, Growth Conditions, and Plant Processing on the Phytochemistry and Antimicrobial Activity of Agrimonia Extracts
Source: Chem Biodivers. 2025 Aug 8;22(12):e01283. doi: 10.1002/cbdv.202501283 (PMC12715982; doi:10.1002/cbdv.202501283)
Supplement: Supplementary file 1 — Supporting file 1: Supporting Information.docx [file CBDV-22-e01283-s001.docx]

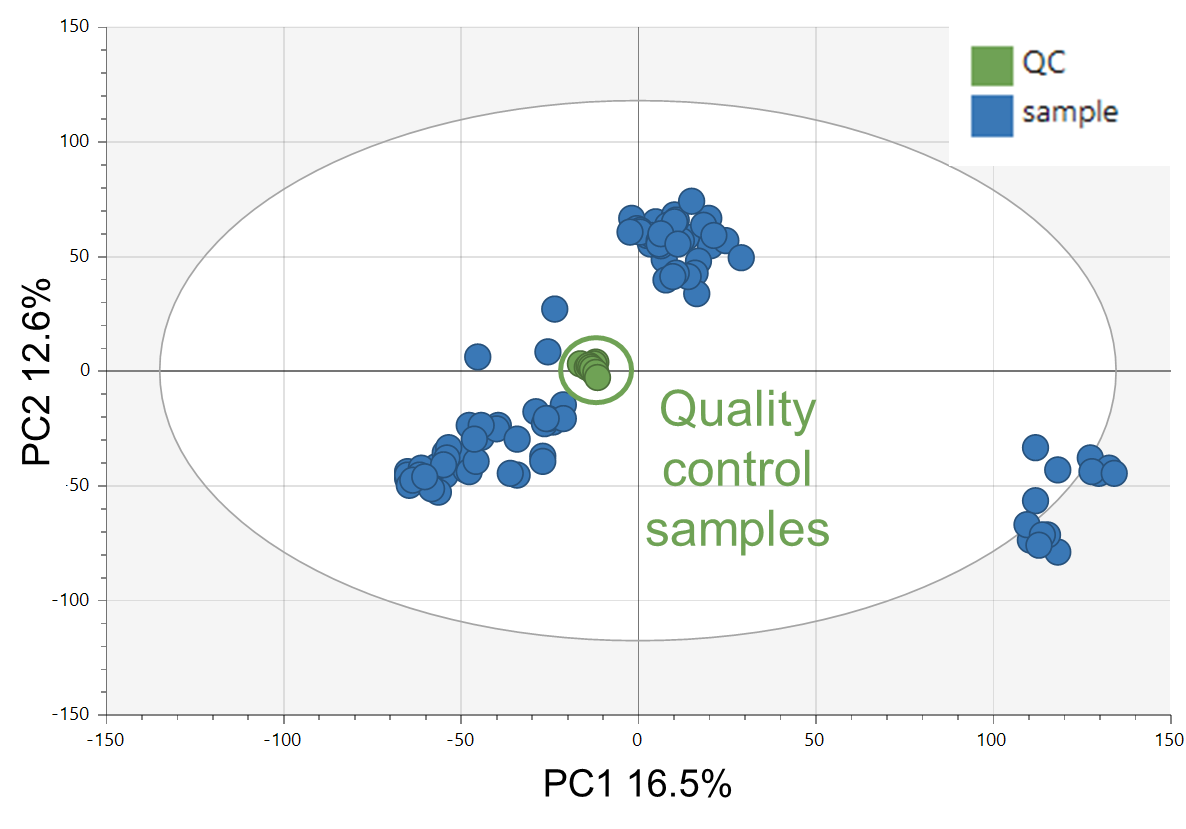


**Figure S1**: Principal component analysis (PCA) score plot (PC1 and PC2) for the entire sample set with highlighted quality control sample as an indicator of measurement stability.

**
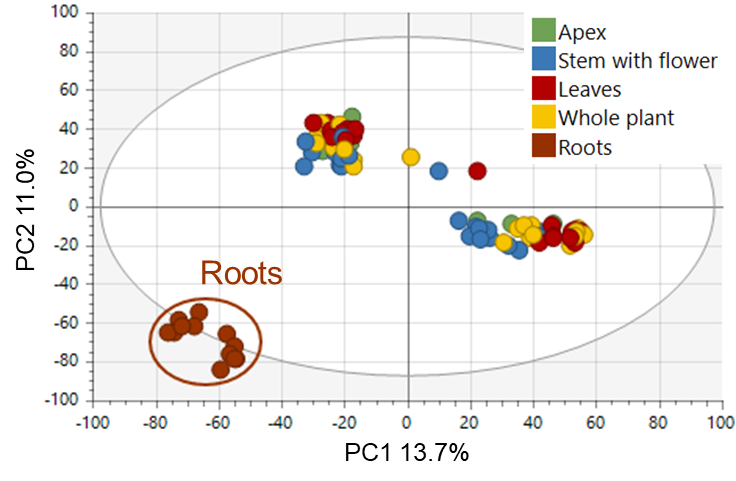
**

**Figure S2**: Principal component analysis (PCA) score plot (PC1 and PC2) of the entire experimental set clustered according to the plant part visualizing the clear distinction of roots samples.

**Table S1**: Antimicrobial activities expressed as IC_50_ in mg/mL of extracts from A. eupatoria from Lednice

| **Species** | **Location** | **Ontogenesis** | **Sample processing** | **Plant part** | ***S. aureus*** | ***E. faecalis*** | ***S. aureus MRSA*** | ***Enterococcus sp. VRE*** | ***P. aeruginosa*** | ***C. albicans*** |
| --- | --- | --- | --- | --- | --- | --- | --- | --- | --- | --- |
| *A. eupatoria* | Lednice | Vegetative | F | A | - | - | - | - | - | 0.42 ± 0.038 ^a,b^ |
|  |  |  | D | A | - | - | - | - | - | - |
|  |  | Beginning of flowering | F | WP | - | 0.23 ±0.020^a^ | - | 0.23 ± 0.008^a,b^ | 0.07 ± 0.008 ^a,b,c^ | 0.32 ± 0.024 ^a,b^ |
|  |  |  |  | S | - | 0.61 ± 0.084^c,d^ | - | - | - | - |
|  |  |  |  | L | 0.28 ± 0.017 ^e,f,g,h,i,j^ | 0.25 ± 0.017^a^ | - | - | - | - |
|  |  |  | D | WP | 0.44 ± 0.033 ^k,l,m,n,o,p,q^ | - | - | 0.20 ± 0.064^a,b^ | - | - |
|  |  |  |  | S | 0.41 ± 0.019 ^j,k,i,m,n,o,p^ | - | - | 0.29 ± 0.050^a,b^ | - | - |
|  |  |  |  | L | 0.56 ± 0.03 ^q,r^ | - | - | 0.23 ± 0.024^a,b^ | - | - |
|  |  | Full bloom | F | WP | - | 0.22 ± 0.001^a^ | - | - | - | - |
|  |  |  |  | S | - | - | - | 0.26 ± 0.040^a,b^ | - | - |
|  |  |  |  | L | - | 0.33 ± 0.077  ^a,b,c^ | - | 0.42 ± 0.221^a,b,c,d^ | - | - |
|  |  |  | D | WP | 0.29 ± 0.009 ^f,g,h,i,j^ | - | - | - | - | - |
|  |  |  |  | S | - | - | - | 0.65 ± 0.161^c,d,e^ | - | - |
|  |  |  |  | L | - | - | - | - | - | - |
|  |  | Senescence | F | R | 0.31 ± 0.010 ^g,h,i,j,k^ | 0.20 ± 0.011^a^ | 0.54 ± 0.008 ^a,b,c,d,e^ | 0.48 ± 0.051^a,b,c,d,e^ | - | - |
|  |  |  | D | R | 0.54 ± 0.035 ^o,p,q,r^ | - | - | 0.74 ± 0.136^d,e^ | - | - |

A – apex, L – leaves, S – stem with flower, WP – whole plant, R – roots, F – fresh-frozen, D – dried

The extracts were applied in the concentration range of 0.01 – 1 mg/mL. The values are presented as the average of three replicates ± standard error of the mean (SEM). Data for IC_50_ were analyzed by one-way analysis of variance (ANOVA) (p ≤ 0.05) with the Tukey's post-hoc test. The means with the same letter within columns are not significantly different at p > 0.05

**Table S2**: Antimicrobial activities expressed as IC_50_ in mg/mL of extracts from A. procera from Lednice

| **Species** | **Location** | **Ontogenesis** | **Sample processing** | **Plant part** | ***S. aureus*** | ***E. faecalis*** | ***S. aureus MRSA*** | ***Enterococcus sp. VRE*** | ***P. aeruginosa*** | ***C. albicans*** |
| --- | --- | --- | --- | --- | --- | --- | --- | --- | --- | --- |
| *A. procera* | Lednice | Vegetative | F | A | - | - | 0.77 ± 0.101 ^c,d,e,f,g^ | - | - | 0.79 ± 0.047^c^ |
|  |  |  | D | A | - | - | - | - | - | - |
|  |  | Beginning of flowering | F | WP | - | - | - | - | - | - |
|  |  |  |  | S | - | - | - | - | - | - |
|  |  |  |  | L | 0.56 ± 0.012 ^q,r^ | - | 0.88 ± 0.046 ^e,f,g^ | - | - | - |
|  |  |  | D | WP | 0.52 ± 0.030 ^n,o,p,q,r^ | - | 0.71 ± 0.100 ^b,c,d,e,f,g^ | - | 0.17 ± 0.022 ^d,e,f,g,h^ | - |
|  |  |  |  | S | - | - | - | - | 0.07 ± 0.006 ^a,b,c^ | - |
|  |  |  |  | L | 0.26 ± 0.003 ^d,e,f,g,h,i^ | - | 0.86 ± 0.016 ^e,f,g^ | - | 0.18 ± 0.010 ^f,g,h^ | - |
|  |  | Full bloom | F | WP | - | - | - | - | - | - |
|  |  |  |  | S | 0.53 ± 0.020 ^n,o,p,q,r^ | - | - | - | - | - |
|  |  |  |  | L | - | - | - | - | 0.04 ± 0.002^a^ | 0.89 ± 0.073^c^ |
|  |  |  | D | WP | 0.54 ± 0.020 ^p,q,r^ | - | - | - | - | - |
|  |  |  |  | S | 0.58 ± 0.070^r^ | - | - | - | - | - |
|  |  |  |  | L | 0.24 ± 0.007 ^d,e,f,g,h^ | - | 0.94 ± 0.033^f,g^ | - | 0.17 ± 0.006^1; e,f,g,h^ | - |
|  |  | Senescence | F | R | 0.10 ± 0.012 ^a,b,c^ | - | - | - | 0.03 ± 0.003^a^ | - |
|  |  |  | D | R | 0.09 ± 0.009 ^a,b,c^ | - | - | - | 0.08 ± 0.003 ^a,b,c,d^ | - |

A – apex, L – leaves, S – stem with flower, WP – whole plant, R – roots, F – fresh-frozen, D – dried

The extracts were applied in the concentration range of 0.01 – 1 mg/mL. The values are presented as the average of three replicates ± standard error of the mean (SEM). Data for IC_50_ were analyzed by one-way analysis of variance (ANOVA) (p ≤ 0.05) with the Tukey's post-hoc test. The means with the same letter within columns are not significantly different at p > 0.05

**Table S3**: Antimicrobial activities expressed as IC_50_ in mg/mL of extracts from A. eupatoria from Hlohovec

| **Species** | **Location** | **Ontogenesis** | **Sample processing** | **Plant part** | ***S. aureus*** | ***E. faecalis*** | ***S. aureus MRSA*** | ***Enterococcus sp. VRE*** | ***P. aeruginosa*** | ***C. albicans*** |
| --- | --- | --- | --- | --- | --- | --- | --- | --- | --- | --- |
| *A. eupatoria* | Hlohovec | Vegetative | F | A | 0.36 ± 0.018 ^h,i,j,k,l^ | 0.35 ± 0.124 ^a,b,c^ | 0.84 ± 0.016 ^e,f,g^ | 0.23 ± 0.034^b^ | 0.09 ± 0.007 ^a,b,c,d,e^ | 0.30 ± 0.009^a,b^ |
|  |  |  | D | A | - | - | 0.86 ± 0.015 ^e,f,g^ | 0.29 ± 0.019^a,b^ | - | 0.43 ± 0.058^a,b^ |
|  |  | Beginning of flowering | F | WP | 0.31 ± 0.022 ^g,h,i,j,k^ | 0.24 ± 0.035^a^ | 0.94 ± 0.025^g^ | 0.21 ± 0.004^a,b^ | - | - |
|  |  |  |  | L | - | 0.30 ± 0.043^a,b^ | 0.58 ± 0.153 ^a,b,c,d,e^ | 0.20 ± 0.011^a,b^ | - | - |
|  |  |  | D | WP | - | 0.68 ± 0.231^d,e^ | - | 0.25 ± 0.011^a,b^ | - | 0.49 ± 0.036^b^ |
|  |  |  |  | S | - | - | - | - | 0.05 ± 0.004^a,b^ | - |
|  |  |  |  | L | 0.30 ± 0.021 ^f,g,h,i,j^ | - | - | - | 0.24 ± 0.013 ^h,i,j^ | - |
|  |  | Full bloom | F | WP | 0.60 ± 0.040 ^r^ | 1.0 ± 0.000^e^ | 0.84 ± 0.105 ^d,e,f,g^ | 0.33 ± 0.007^a,b,c^ | - | - |
|  |  |  |  | L | - | - | 0.52 ± 0.032 ^a,b,c,d,e^ | 0.50 ± 0.047^a,b,c,d,e^ | - | - |
|  |  |  | D | WP | 0.30 ± 0.015 ^f,g,h,I,j^ | - | 0.7 ± 0.071 ^a,b,c,d,e,f,g^ | 0.58 ± 0.126^b,c,d,e^ | 0.23 ± 0.006 ^g,h,i,j^ | - |
|  |  |  |  | S | - | - | - | 0.44 ± 0.057^a,b,c,d^ | 0.23 ± 0.018 ^g,h,i,j^ | - |
|  |  |  |  | L | - | - | 0.83 ± 0.011 ^c,d,e,f,g^ | 0.45 ± 0.184^a,b,c,d^ | - | - |
|  |  | Senescence | F | R | 0.28 ± 0.010 ^e,f,g,h,I,j^ | 0.28 ± 0.036^a,b^ | 0.46 ± 0.008 ^a,b,c,d^ | 0.24 ± 0.004^a,b^ | - | - |
|  |  |  | D | R | 0.40 ± 0.050 ^i,j,k,l,m,n^ | - | - | 0.53 ± 0.021^a,b,c,d,e^ | 0.18 ± 0.004 ^f,g,h^ | - |

A – apex, L – leaves, S – stem with flower, WP – whole plant, R – roots, F – fresh-frozen, D – dried

The extracts were applied in the concentration range of 0.01 – 1 mg/mL. The values are presented as the average of three replicates ± standard error of the mean (SEM). Data for IC_50_ were analyzed by one-way analysis of variance (ANOVA) (p ≤ 0.05) with the Tukey's post-hoc test. The means with the same letter within columns are not significantly different at p > 0.05

**Table S4**: Antimicrobial activities expressed as IC_50_ in mg/mL of extracts from A. eupatoria from Milovice

| **Species** | **Location** | **Ontogenesis** | **Sample processing** | **Plant part** | ***S. aureus*** | ***E. faecalis*** | ***S. aureus MRSA*** | ***Enterococcus sp. VRE*** | ***P. aeruginosa*** | ***C. albicans*** |
| --- | --- | --- | --- | --- | --- | --- | --- | --- | --- | --- |
| *A. eupatoria* | Milovice | Vegetative | F | A | - | - | - | - | - | 0.85 ± 0.055^c^ |
|  |  |  | D | A | - | - | - | - | - | - |
|  |  | Beginning of flowering | F | WP | - | 0.22 ± 0.029^a^ | - | - | - | - |
|  |  |  |  | S | - | 0.58 ± 0.154 ^b,c,d^ | - | - | - | - |
|  |  |  |  | L | - | - | - | - | - | 0.74 ± 0.001^c^ |
|  |  |  | D | WP | 0.40 ± 0.023 ^i,j,k,l,m,n,o^ | - | - | - | - | - |
|  |  |  |  | S | - | - | - | - | - | - |
|  |  |  |  | L | 0.86 ± 0.105 ^s^ | - | - | - | - | - |
|  |  | Full bloom | F | WP | - | - | - | - | - | 0.31 ± 0.023^a,b^ |
|  |  |  |  | S | - | - | - | - | - | 0.33 ± 0.020^a,b^ |
|  |  |  |  | L | - | - | - | - | - | - |
|  |  |  | D | WP | - | - | - | - | - | - |
|  |  |  |  | S | 0.59 ± 0.060 ^r^ | - | - | - | - | - |
|  |  |  |  | L | - | - | - | - | - | - |
|  |  | Senescence | F | R | 0.27 ± 0.024 ^d,e,f,g,h,i^ | - | 0.37 ± 0.001^a,b^ | 0.39 ± 0.107^a,b,c,d^ | 0.13 ± 0.000 ^b,c,d,e,f^ | - |
|  |  |  | D | R | 0.57 ± 0.005 ^q,r^ | - | - | 0.76 ± 0.214^d,e^ | 0.04 ± 0.002^a^ | - |

A – apex, L – leaves, S – stem with flower, WP – whole plant, R – roots, F – fresh-frozen, D – dried

The extracts were applied in the concentration range of 0.01 – 1 mg/mL. The values are presented as the average of three replicates ± standard error of the mean (SEM). Data for IC_50_ were analyzed by one-way analysis of variance (ANOVA) (p ≤ 0.05) with the Tukey's post-hoc test. The means with the same letter within columns are not significantly different at p > 0.05

**Table S5**: Antimicrobial activities expressed as IC_50_ in mg/mL of extracts from A. procera from Olomouc

| **Species** | **Location** | **Ontogenesis** | **Sample processing** | **Plant part** | ***S. aureus*** | ***E. faecalis*** | ***S. aureus MRSA*** | ***Enterococcus sp. VRE*** | ***P. aeruginosa*** | ***C. albicans*** |
| --- | --- | --- | --- | --- | --- | --- | --- | --- | --- | --- |
| *A. procera* | Olomouc | Vegetative | F | A | - | - | - | - | - | 0.23 ± 0.010^a^ |
|  |  |  | D | A | 0.27 ± 0.033 ^d,e,f,g,h,i^ | - | 0.76 ± 0.151 ^c,d,e,f,g^ | - | 0.36 ± 0.043^k^ | - |
|  |  | Beginning of flowering | F | WP | 0.26 ± 0.007 ^d,e,f,g,h,i^ | - | 0.96 ± 0.031^g^ | - | - | - |
|  |  |  |  | S | - | - | - | - | - | 0.78 ± 0.111^c^ |
|  |  |  |  | L | 0.20 ± 0.006 ^b,c,d,e,f,g^ | - | 0.97 ± 0.004^g^ | - | - | - |
|  |  |  | D | WP | 0.16 ± 0.012 ^a,b,c,d,e,f^ | - | 0.52 ± 0.045 ^a,b,c,d,e^ | - | - | - |
|  |  |  |  | S | - | 0.77 ± 0.099^d,e^ | - | 0.81 ± 0.092^e^ | - | - |
|  |  |  |  | L | 0.07 ± 0.003 ^a,b^ | - | 0.36 ± 0.014^a^ | - | - | - |
|  |  | Full bloom | F | WP | 0.60 ± 0.040 ^r^ | - | - | - | - | - |
|  |  |  |  | S | 0.52 ± 0.044 ^n,o,p,q,r^ | - | - | - | - | - |
|  |  |  |  | L | 0.37 ± 0.050 ^h,i,j,k,l,m^ | - | - | - | - | - |
|  |  |  | D | WP | 0.30 ± 0.015 ^f,g,h,i,j^ | - | 0.73 ± 0.090 ^a,b,c,d,e,f,g^ | - | 0.19 ± 0.020 ^f,g,h^ | - |
|  |  |  |  | S | 0.51 ± 0.005 ^m,n,o,p,q,r^ | - | - | - | 0.24 ± 0.016 ^h,i,j^ | - |
|  |  |  |  | L | 0.30 ± 0.006 ^f,g,h,i,j^ | - | - | - | 0.21 ± 0.012 ^f,g,h,i^ | - |
|  |  | Senescence | F | R | 0.07 ± 0.007 ^a,b^ | - | 0.44 ± 0.005 ^a,b,c^ | 0.24 ± 0.009^a,b^ | 0.02 ± 0.002^a^ | - |
|  |  |  | D | R | 0.04 ± 0.001 ^a^ | - | 0.96 ± 0.011^g^ | - | 0.02 ± 0.002^a^ | - |

A – apex, L – leaves, S – stem with flower, WP – whole plant, R – roots, F – fresh-frozen, D – dried

The extracts were applied in the concentration range of 0.01 – 1 mg/mL. The values are presented as the average of three replicates ± standard error of the mean (SEM). Data for IC_50_ were analyzed by one-way analysis of variance (ANOVA) (p ≤ 0.05) with the Tukey's post-hoc test. The means with the same letter within columns are not significantly different at p > 0.05

**Table S6**: Antimicrobial activities expressed as IC_50_ in mg/mL of extracts from A. procera from Olomouc

| **Species** | **Location** | **Ontogenesis** | **Sample processing** | **Plant part** | ***S. aureus*** | ***E. faecalis*** | ***S. aureus MRSA*** | ***Enterococcus sp. VRE*** | ***P. aeruginosa*** | ***C. albicans*** |
| --- | --- | --- | --- | --- | --- | --- | --- | --- | --- | --- |
| *A. procera* | Olomouc | Vegetative | F | A | 0.32 ± 0.061 ^h,i,j,k,l,m^ | - | - | - | - | - |
|  |  |  | D | A | 0.14 ± 0.015 ^a,b,c,d^ | - | - | 0.33 ± 0.154^a,b,c^ | - | - |
|  |  | Beginning of flowering | F | WP | 0.28 ± 0.02 ^e,f,g,h,I,j^ | - | 0.86 ± 0.015 ^e,f,g^ | - | - | - |
|  |  |  |  | S | - | - | - | - | - | - |
|  |  |  |  | L | 0.27 ± 0.012 ^d,e,f,g,h,i^ | - | 0.87 ± 0.018 ^e,f,g^ | - | - | - |
|  |  |  | D | WP | 0.44 ± 0.023 ^k,l,m,n,o,p,q^ | - | 0.70 ± 0.111 ^a,b,c,d,e,f,g^ | - | 0.29 ± 0.007 ^i,j,k^ | - |
|  |  |  |  | S | - | - | - | - | - | - |
|  |  |  |  | L | 0.29 ± 0.022 ^f,g,h,u,j^ | - | - | - | 0.29 ± 0.009^j,k^ | - |
|  |  | Full bloom | F | WP | 0.47 ± 0.062 ^l,m,n,o,p,q,r^ | - | - | - | - | - |
|  |  |  |  | S | - | - | - | - | - | - |
|  |  |  |  | L | 0.21 ± 0.007 ^c,d,e,f,g^ | - | - | - | 0.15 ± 0.006 ^c,d,e,f,g^ | - |
|  |  |  | D | WP | 0.54 ± 0.005 ^o,p,q,r^ | - | - | - | 0.36 ± 0.038^k^ | - |
|  |  |  |  | S | - | - | - | - | - | - |
|  |  |  |  | L | 0.15 ± 0.012 ^a,b,c,d,e^ | - | - | - | 0.04 ± 0.003^a^ | - |
|  |  | Senescence | F | R | 0.05 ± 0.001 ^a^ | - | 0.38 ± 0.004^a,b^ | 0.24 ± 0.006^a,b^ | 0.03 ± 0.003^a^ | - |
|  |  |  | D | R | 0.20 ± 0.010 ^b,c,d,e,f,g^ | - | - | - | 0.17 ± 0.009 ^f,g,h^ | - |

A – apex, L – leaves, S – stem with flower, WP – whole plant, R – roots, F – fresh-frozen, D – dried

The extracts were applied in the concentration range of 0.01 – 1 mg/mL. The values are presented as the average of three replicates ± standard error of the mean (SEM). Data for IC_50_ were analyzed by one-way analysis of variance (ANOVA) (p ≤ 0.05) with the Tukey's post-hoc test. The means with the same letter within columns are not significantly different at p > 0.05

**Table S7**: Taxonomic verification of Agrimonia samples

| **No.** | **Identification** | **Place of collection** | **Origin** | **Inventory** | **Herbarium code** |
| --- | --- | --- | --- | --- | --- |
| S1 | *A. eupatoria* | Lednice | cultivated | B 3350 | 111/2025 |
| S2 | *A. procera* | Lednice | cultivated | B 3351 | 111/2025 |
| S3 | *A. eupatoria* | Hlohovec | cultivated | B 3352 | 111/2025 |
| S4 | *A. eupatoria* | Milovice | wild | B 3353 | 111/2025 |
| S5^a^ | *A. procera* | Olomouc | cultivated | 09A0500004 | - |
| S6^b^ | *A. procera* | Olomouc | cultivated | 09A0500001 | - |

^a^ The plant sample initially assumed to represent A*. eupatoria* was taxonomically reclassified as *A. procera* based on evidence from UHPLC-HRMS/MS analysis and statistical evaluation (see the Experimental section of the main article for further details).

^b^ The cultivar of *A. procera* WALLR., which is historically misclassified as *A. eupatoria* L. ^[46]^

***
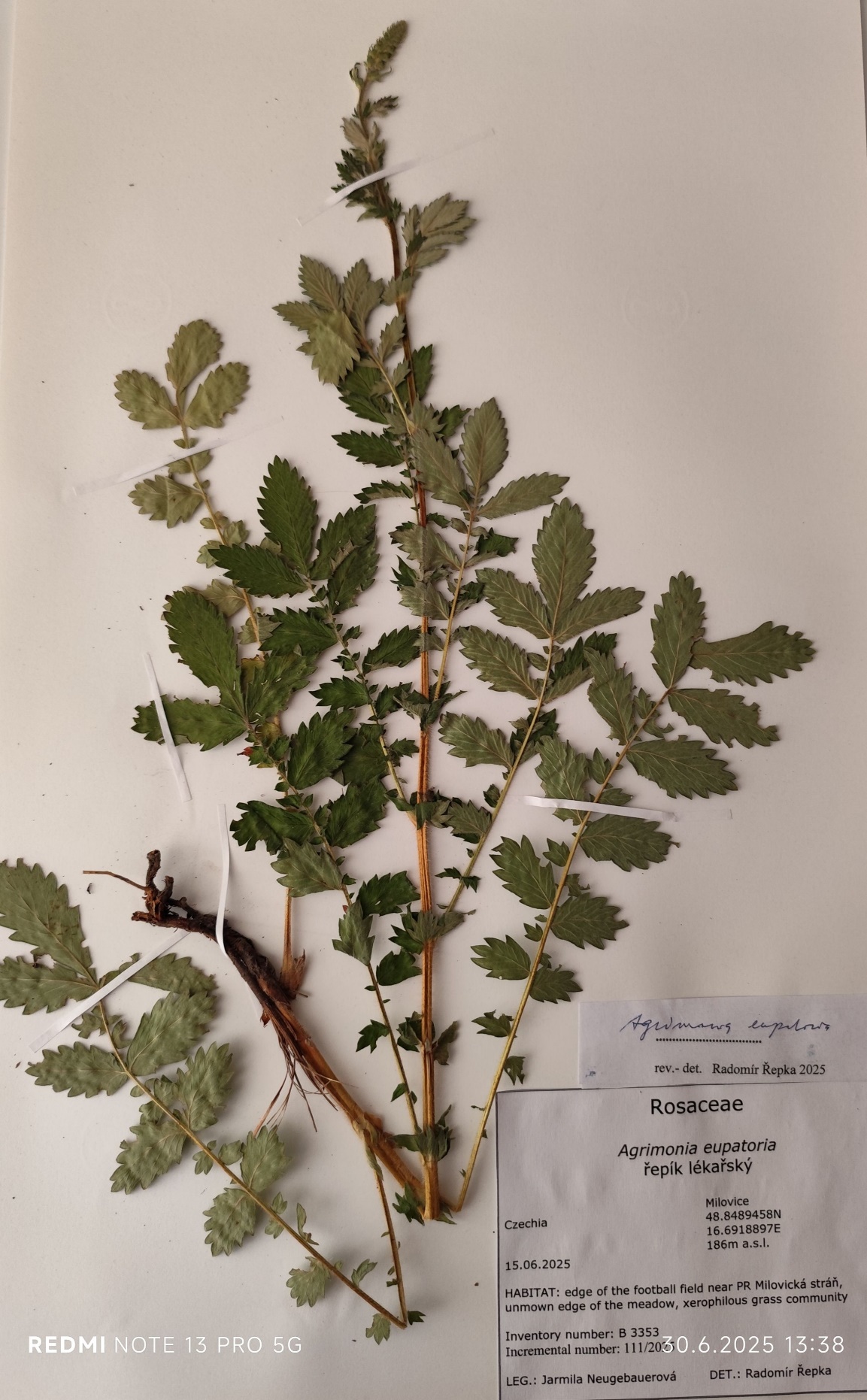
***

**Figure S3**: Herbarium item of wild *A. eupatoria* collected in the locality of Milovice (*Agrimonia* sample numbered as S4 in the **Table S7**)

**Table S8**: Agrimonia sample collection, 2021

| **Sampling date** | **Cultivated**  **/ Wild** | **Locality** | **Variety** | **Ontogenesis phase** | **Plant**  **part** | **Post-harvest**  **sample processing** | **Extraction ratio** |
| --- | --- | --- | --- | --- | --- | --- | --- |
| 25.05.2021 | C | Lednice | *Eupatoria* | Vegetative | Apex | Fresh frozen (-18°C) | 1:5 |
|  |  |  |  |  |  | Dried | 1:5 |
| 25.05.2021 | C | Lednice | *Procera* | Vegetative | Apex | Fresh frozen (-18°C) | 1:7.5 |
|  |  |  |  |  |  | Dried | 1:5 |
| 25.05.2021 | C | Hlohovec | *Eupatoria* | Vegetative | Apex | Fresh frozen (-18°C) | 1:5 |
|  |  |  |  |  |  | Dried | 1:6.25 |
| 26.05.2021 | W | Milovice | *Eupatoria* | Vegetative | Apex | Fresh frozen (-18°C) | 1:5 |
|  |  |  |  |  |  | Dried | 1:6.25 |
| 28.05.2021 | C | Olomouc | *Procera* | Vegetative | Apex | Fresh frozen (-18°C) | 1:5 |
|  |  |  |  |  |  | Dried | 1:5 |
| 28.05.2021 | C | Olomouc | *Procera* | Vegetative | Apex | Fresh frozen (-18°C) | 1:5 |
|  |  |  |  |  |  | Dried | 1:7.5 |
| 22.06.2021 | C | Lednice | *Eupatoria* | Beginning  of flowering | Whole plant | Fresh frozen (-18°C) | 1:5 |
|  |  |  |  |  | Stem with flower | Fresh frozen (-18°C) | 1:5 |
|  |  |  |  |  | Leaves | Fresh frozen (-18°C) | 1:5 |
|  |  |  |  |  | Whole plant | Dried | 1:5 |
|  |  |  |  |  | Stem with flower | Dried | 1:5 |
|  |  |  |  |  | Leaves | Dried | 1:15 |
| 22.06.2021 | C | Lednice | *Procera* | Beginning  of flowering | Whole plant | Fresh frozen (-18°C) | 1:5 |
|  |  |  |  |  | Stem with flower | Fresh frozen (-18°C) | 1:5 |
|  |  |  |  |  | Leaves | Fresh frozen (-18°C) | 1:5 |
|  |  |  |  |  | Whole plant | Dried | 1:5 |
|  |  |  |  |  | Stem with flower | Dried | 1:5 |
|  |  |  |  |  | Leaves | Dried | 1:5 |
| 22.06.2021 | C | Hlohovec | *Eupatoria* | Beginning  of flowering | Whole plant | Fresh frozen (-18°C) | 1:5 |
|  |  |  |  |  | Leaves | Fresh frozen (-18°C) | 1:5 |
|  |  |  |  |  | Whole plant | Dried | 1:6.25 |
|  |  |  |  |  | Stem with flower | Dried | 1:5 |
|  |  |  |  |  | Leaves | Dried | 1:7.5 |
| 22.06.2021 | W | Milovice | *Eupatoria* | Beginning  of flowering | Whole plant | Fresh frozen (-18°C) | 1:5 |
|  |  |  |  |  | Stem with flower | Fresh frozen (-18°C) | 1:5 |
|  |  |  |  |  | Leaves | Fresh frozen (-18°C) | 1:5 |
|  |  |  |  |  | Whole plant | Dried | 1:6.25 |
|  |  |  |  |  | Stem with flower | Dried | 1:5 |
|  |  |  |  |  | Leaves | Dried | 1:10 |
| 23.06.2021 | C | Olomouc | *Procera* | Beginning  of flowering | Whole plant | Fresh frozen (-18°C) | 1:5 |
|  |  |  |  |  | Stem with flower | Fresh frozen (-18°C) | 1:5 |
|  |  |  |  |  | Leaves | Fresh frozen (-18°C) | 1:5 |
|  |  |  |  |  | Whole plant | Dried | 1:5 |
|  |  |  |  |  | Stem with flower | Dried | 1:5 |
|  |  |  |  |  | Leaves | Dried | 1:5 |
| 23.06.2021 | C | Olomouc | *Procera* | Beginning  of flowering | Whole plant | Fresh frozen (-18°C) | 1:5 |
|  |  |  |  |  | Stem with flower | Fresh frozen (-18°C) | 1:5 |
|  |  |  |  |  | Leaves | Fresh frozen (-18°C) | 1:5 |
|  |  |  |  |  | Whole plant | Dried | 1:5 |
|  |  |  |  |  | Stem with flower | Dried | 1:5 |
|  |  |  |  |  | Leaves | Dried | 1:5 |

**Table S8**: Agrimonia sample collection, 2021 - continuation

| **Sampling date** | **Cultivated**  **/ Wild** | **Locality** | **Variety** | **Ontogenesis phase** | **Plant**  **Part** | **Post-harvest**  **Sample processing** | **Extraction ratio** |
| --- | --- | --- | --- | --- | --- | --- | --- |
| 08.07.2021 | C | Lednice | *Eupatoria* | Full bloom | Whole plant | Fresh frozen (-18°C) | 1:5 |
|  |  |  |  |  | Stem with flower | Fresh frozen (-18°C) | 1:5 |
|  |  |  |  |  | Leaves | Fresh frozen (-18°C) | 1:5 |
|  |  |  |  |  | Whole plant | Dried | 1:5 |
|  |  |  |  |  | Stem with flower | Dried | 1:5 |
|  |  |  |  |  | Leaves | Dried | 1:7.5 |
| 08.07.2021 | C | Lednice | *Procera* | Full bloom | Whole plant | Fresh frozen (-18°C) | 1:5 |
|  |  |  |  |  | Stem with flower | Fresh frozen (-18°C) | 1:5 |
|  |  |  |  |  | Leaves | Fresh frozen (-18°C) | 1:5 |
|  |  |  |  |  | Whole plant | Dried | 1:6.25 |
|  |  |  |  |  | Stem with flower | Dried | 1:5 |
|  |  |  |  |  | Leaves | Dried | 1:7.5 |
| 07.07.2021 | C | Hlohovec | *Eupatoria* | Full bloom | Whole plant | Fresh frozen (-18°C) | 1:5 |
|  |  |  |  |  | Leaves | Fresh frozen (-18°C) | 1:5 |
|  |  |  |  |  | Whole plant | Dried | 1:5 |
|  |  |  |  |  | Stem with flower | Dried | 1:5 |
|  |  |  |  |  | Leaves | Dried | 1:7.5 |
| 07.07.2021 | W | Milovice | *Eupatoria* | Full bloom | Whole plant | Fresh frozen (-18°C) | 1:5 |
|  |  |  |  |  | Stem with flower | Fresh frozen (-18°C) | 1:5 |
|  |  |  |  |  | Leaves | Fresh frozen (-18°C) | 1:5 |
|  |  |  |  |  | Whole plant | Dried | 1:5 |
|  |  |  |  |  | Stem with flower | Dried | 1:5 |
|  |  |  |  |  | Leaves | Dried | 1:7.5 |
| 08.07.2021 | C | Olomouc | *Procera* | Full bloom | Whole plant | Fresh frozen (-18°C) | 1:5 |
|  |  |  |  |  | Stem with flower | Fresh frozen (-18°C) | 1:5 |
|  |  |  |  |  | Leaves | Fresh frozen (-18°C) | 1:5 |
|  |  |  |  |  | Whole plant | Dried | 1:5 |
|  |  |  |  |  | Stem with flower | Dried | 1:5 |
|  |  |  |  |  | Leaves | Dried | 1:5 |
| 08.07.2021 | C | Olomouc | Procera | Full bloom | Whole plant | Fresh frozen (-18°C) | 1:5 |
|  |  |  |  |  | Stem with flower | Fresh frozen (-18°C) | 1:5 |
|  |  |  |  |  | Leaves | Fresh frozen (-18°C) | 1:5 |
|  |  |  |  |  | Whole plant | Dried | 1:5 |
|  |  |  |  |  | Stem with flower | Dried | 1:5 |
|  |  |  |  |  | Leaves | Dried | 1:5 |
| 11.10.2021 | C | Lednice | *Eupatoria* | Senescence | Roots | Fresh frozen (-18°C) | 1:5 |
|  |  |  |  |  |  | Dried | 1:5 |
| 11.10.2021 | C | Lednice | *Procera* | Senescence | Roots | Fresh frozen (-18°C) | 1:5 |
|  |  |  |  |  |  | Dried | 1:5 |
| 11.10.2021 | C | Hlohovec | *Eupatoria* | Senescence | Roots | Fresh frozen (-18°C) | 1:5 |
|  |  |  |  |  |  | Dried | 1:5 |
| 10.10.2021 | W | Milovice | *Eupatoria* | Senescence | Roots | Fresh frozen (-18°C) | 1:5 |
|  |  |  |  |  |  | Dried | 1:5 |
| 08.10.2021 | C | Olomouc | *Procera* | Senescence | Roots | Fresh frozen (-18°C) | 1:5 |
|  |  |  |  |  |  | Dried | 1:5 |
| 08.10.2021 | C | Olomouc | *Procera* | Senescence | Roots | Fresh frozen (-18°C) | 1:5 |
|  |  |  |  |  |  | Dried | 1:5 |
